# Integrating structure-from-motion photogrammetry with geospatial software as a novel technique for quantifying 3D ecological characteristics of coral reefs

**DOI:** 10.7717/peerj.1077

**Published:** 2015-07-07

**Authors:** JHR Burns, D Delparte, RD Gates, M Takabayashi

**Affiliations:** 1 Department of Biology, College of Natural Sciences, University of Hawaiʻi at Mānoa, McCarthy Mall, Honolulu, HI, USA; 2 Department of Geosciences, Idaho State University, Pocatello, ID, USA; 3 Hawaiʻi Institute of Marine Biology, University of Hawaiʻi at Mānoa, Kaneohe, HI, USA; 4 Marine Science Department, University of Hawaiʻi at Hilo, Hilo, HI, USA

**Keywords:** Coral, Coral reef, Reef structural complexity, 3D topographic reconstruction, Coral ecology, Photogrammetry, Habitat, Structure-from-motion

## Abstract

The structural complexity of coral reefs plays a major role in the biodiversity, productivity, and overall functionality of reef ecosystems. Conventional metrics with 2-dimensional properties are inadequate for characterization of reef structural complexity. A 3-dimensional (3D) approach can better quantify topography, rugosity and other structural characteristics that play an important role in the ecology of coral reef communities. Structure-from-Motion (SfM) is an emerging low-cost photogrammetric method for high-resolution 3D topographic reconstruction. This study utilized SfM 3D reconstruction software tools to create textured mesh models of a reef at French Frigate Shoals, an atoll in the Northwestern Hawaiian Islands. The reconstructed orthophoto and digital elevation model were then integrated with geospatial software in order to quantify metrics pertaining to 3D complexity. The resulting data provided high-resolution physical properties of coral colonies that were then combined with live cover to accurately characterize the reef as a living structure. The 3D reconstruction of reef structure and complexity can be integrated with other physiological and ecological parameters in future research to develop reliable ecosystem models and improve capacity to monitor changes in the health and function of coral reef ecosystems.

## Introduction

The calcium carbonate skeletons deposited by reef forming corals provide the structural framework for highly diverse and productive coral reef ecosystems throughout tropical and subtropical oceans. These coastal ecosystems are among the most ecologically and economically productive on the planet, providing goods and services to humans through fisheries, coastline protection, building materials, biochemical compounds, and tourism (Moberg & Folke, 1999; Hoegh-Guldberg et al., 2007). Coral reefs are extremely vulnerable to stress and many have declined in integrity in the face of the increasing intensity and frequency of disturbances associated with climate change and human use (Harvell et al., 1999; Hoegh-Guldberg et al., 2007). Against this backdrop, it is critical to accurately evaluate and better understand how the structural integrity and ecological processes of these ecosystems respond to environmental changes.

Reef structural framework and reef-building capacity of biogenic components are primary drivers of the coral reef ecosystem functionality (Done, 1997; Fisher et al., 2007; Alvarez-Filip et al., 2009; Zawada, Piniak & Hearn, 2010). These factors have a reciprocal relationship with one another: the high level of architectural complexity provides a diverse range of microhabitats that support high biodiversity, productivity, and resilience; in turn, biogenic calcification is responsible for the reef-building processes that create the complex physical structure (Risk, 1972; Luckhurst & Luckhurst, 1978; Crowder & Cooper, 1982; Idjadi et al., 2006; Wilson, Graham & Polunin, 2007; Goatley & Bellwood, 2011; Graham & Nash, 2013). Furthermore, the extraordinary reef building capacity of scleractinian corals, enabled by their symbiotic partner *Symbiodinium*, is greatly influenced by physical characteristics of coral colonies. This is because physical characteristics of coral colonies like overall shape and topographic complexity influence key metabolic processes such as photosynthesis, respiration, calcium carbonate deposition, and reproduction (Smith, 1978; Crossland, Hatcher & Smith, 1991; Fisher et al., 2007). An accurate evaluation of the physical characteristics of coral colonies and coral reefs, involving 3-dimentional (3D) metrics, is critical in advancing our understanding of functionality of not only coral biology but also habitat availability, biogenic flux, and productivity of the entire coral reef ecosystem (Goatley & Bellwood, 2011; Pittman & Brown, 2011; Graham & Nash, 2013).

Recent advances in the fields of computer vision and photogrammetry, as well as improvements in data processing power, now make generation of 3D models and orthophotos from 2D imagery cost- and time-effective. Historically, collecting 3D environmental data has been a challenging undertaking in terms of logistics and cost. Oceanographic remote sensing tools, such as light detection and ranging (LIDAR), have been mounted to vessels for long-rage 3D underwater imaging, and in recent years, researchers have developed new techniques for high-resolution 3D reconstruction and visualization of underwater habitats using robotic vehicles (Kocak & Caimi, 2005; Pizarro, Eustice & Singh, 2009; Johnson-Roberson et al., 2010). Most of these technologies have not been widely used for small-scale coral reef studies due to the complexity of data collection, analytical time required, and prohibitive cost (Abdo et al., 2006; Bruno et al., 2011). The more recently developed Structure-from-Motion (SfM) photogrammetric technology offers a simpler, faster, and more affordable alternative for high-resolution topographic reconstruction (Westoby et al., 2012; McCarthy & Benjamin, 2014).

The SfM techniques have improved the quality of 3D data than can be derived from overlapping imagery by incorporating advancements in soft-copy triangulation and image-based terrain extraction algorithms (Westoby et al., 2012). Furthermore, SfM can accurately reconstruct scene geometry using high-resolution overlapping imagery obtained with single lens reflex (SLR) cameras and consumer point-and-shoot cameras, rather then relying on stereoscopic cameras, thus enhancing the accessibility and accuracy of 3D photogrammetric modeling for an array of uses. SfM techniques utilize the basic principles of stereoscopic photogrammetry, which is the science of obtaining reliable information about physical objects, the environment and terrain through processes of recording, measuring, and interpreting photographs or other images. However, the fundamental advantage of SfM is that the geometry of the photographed scene, camera positions, and orientation are evaluated without the need for a priori specification of targets with known 3D positions (Snavely, Seitz & Szeliski, 2008; Westoby et al., 2012). Rather, SfM photogrammetry determines these parameters simultaneously with a highly redundant and iterative bundle adjustment procedure, which is based on a dataset of invariant features extracted from multiple overlapping images (Snavely, Seitz & Szeliski, 2008; Westoby et al., 2012). These features are tracked from image to image, enabling initial estimates of camera position and object coordinates which are then refined iteratively using non-linear least squares minimization (Westoby et al., 2012; Fonstad et al., 2013). This process produces a point cloud of identifiable features present in the input photographs. Once georeferenced, this point cloud can be used to generate an array of digital elevation metrics to quantify 3D characteristics (Fonstad et al., 2013). Automating the process from identification of control points to 3D reconstruction of scene geometry makes SfM substantially more practical and cost-effective than traditional photogrammetric methodologies. Multiple studies have validated the accuracy of SfM techniques for high-resolution 3D topographic reconstruction and analysis, and in some cases found SfM to be highly comparable to substantially more expensive LIDAR techniques (Harwin & Lucieer, 2012; Delparte et al., 2014; Javernick, Brasington & Caruso, 2014).

Underwater SfM 3D reconstruction techniques have primarily been utilized for seafloor habitat characterization, bathymetry mapping, marine environment inspections and archeological surveys (Singh et al., 2002; Pizarro, Eustice & Singh, 2009; Beall et al., 2010; McCarthy & Benjamin, 2014). However, the SfM technology can also be applied to obtain a range of metrics pertaining to coral colony volume, biomass, and live surface area, all of which directly govern biological functions and ecology of corals. SfM photogrammetry also produces digital elevation models (DEMs) that can be analyzed using topographic software tools. This process enables quantification of intricate structural metrics such as surface complexity (3D/2D surface area), slope, and curvature. These 3D structural metrics are important predictors of organismal abundance, biomass and diversity, and also affect benthic current velocities associated with the food particle supply for suspension feeding corals (Kostylev et al., 2005; Guinan et al., 2009; Pittman, Costa & Battista, 2009; Pittman & Brown, 2011). The ability to quantify these topographic features will greatly enhance both biological and ecological investigations of coral reef ecosystems.

This study details a suite of methods for quantifying 3D characteristics of a coral reef using SfM photogrammetry in conjunction with geospatial software tools. This novel approach enables the extraction and quantification of biologically and ecologically meaningful data from 3D reconstructions made with SfM techniques. We tested the described methods at a study site in French Frigate Shoals, the largest atoll in the Papahānaumokuākea Marine National Monument (Northwestern Hawaiian Islands). The objectives of this study were to: (1) create a 3D reconstruction of the benthic habitat; (2) quantify several metrics pertaining to ecologically meaningful 3D characteristics; and (3) determine if each benthic component in the surveyed habitat will exhibit significantly distinct 3D structural characteristics.

## Methods

### Image acquisition of a coral reef at French Frigate Shoals

Images of the coral reef were collected at French Frigate Shoals (23°43′03.2″N, 166°11′10.2″W), an atoll located in the Papahānaumokuākea Marine National Monument. Image collections were conducted in conjunction with annual benthic monitoring surveys performed during a 2012 research expedition aboard *NOAA Ship Hi‘ialakai* (R 334, National Oceanic and Atmospheric Administration, National Ocean Sciences, Permit # PMNM-2012-031). Survey locations were chosen using a random stratified design in order to objectively assess benthic characteristics throughout the Monument. The data presented here were acquired during a single benthic survey at site FFS33 (23°50′11.76″, −166°16′0.48″), at 13:00 on August 5th, 2012. Two divers using SCUBA conducted the image acquisition for SfM analysis at the pre-determined survey location. A transect tape was deployed on the substrate in a rectangular pattern in order to create a 25 × 6-m grid. Polyvinyl reflectors were used as ground control points (GCPs) and placed at the corners and midpoints of the grid in order to have GCPs with known depths and relative spatial locations for accurate *x*, *y*, *z* reference coordinates. The positions and relative distances among the GCPs were recorded using the transect tape and depth values were obtained with a submersible depth gauge. A GPS coordinate was taken for the GCP placed at the start of the survey and the bearings between all GCPs were recorded to enable accurate georeferencing of the survey area. A single calibration grid and two scale markers were also placed in the survey area to validate accuracy and precision of the resulting model. Photographs were taken continuously while swimming in a boustrophodonic pattern above the 25 × 6-m grid at a depth of 3-m above the substrate. The photograph acquisition and the deployment and retrieval of GCPs, scale markers and calibration grid could be completed within 20 minutes, which allowed ample time for a standard visual benthic survey to be completed during the same 60-minute dive. Camera calibration and optimization were completed using the Agisoft PhotoScan (Agisoft LLC., St. Petersburg, Russia) modeling software, which is capable of resolving the optical characteristics of the camera lens directly from the image metadata and performing calibration using Brown’s distortion model. Pre-calibration of the camera in the surveyed environment is desirable, but was not practical for this study because the process requires calibration equipment and substantial time (McCarthy & Benjamin, 2014). When using a hemispheric dome port, the distortion occurring in the underwater environment is radial and the Photoscan software is capable of resolving the optical characteristics of the lens directly from the images without prior calibration, thus eliminating the significant burden of pre-calibration (McCarthy & Benjamin, 2014). Pre-calibration options for the camera should be considered when using SfM approaches, and utilized if feasible for the research approach, as this can improve model accuracy and resolution. The workflow presented here allows for rapid surveys and reduces bulky equipment, which is critical for research conducted in remote locations where time and equipment are limiting factors. All photos were taken using a Pentax K-5 digital SLR camera with a 18–55 mm lens (PENTAX RICOH Imaging Americas Corporation, USA) in an Ikelite housing (Ikelite Underwater Systems, Indianapolis, Indiana, USA). An 8-inch hemispheric dome port was used for the Ikelite housing as this has been shown to significantly reduce refraction and enhance the ability of the software to accurately align the images (Bruno et al., 2011; McCarthy & Benjamin, 2014). Strobes were not used in order to reduce the effect of light changes in shadows. DLSR cameras are preferable for the approach described here as the large sensors and high-quality interchangeable lenses provide better light sensitivity with less noise than compared to small sensor compact cameras. The camera lens was set to the focal length of 18 mm with a shutter speed of 1/180, an aperture of f/8 and an ISO of 800. The environmental conditions of a study site, specifically light availability and turbidity, must be considered when selecting the camera and lens settings as an improper depth of field and lack of light sensitivity can dramatically impair the resolution and precision of underwater photogrammetry.

### 3D model generation

**Figure 1:**
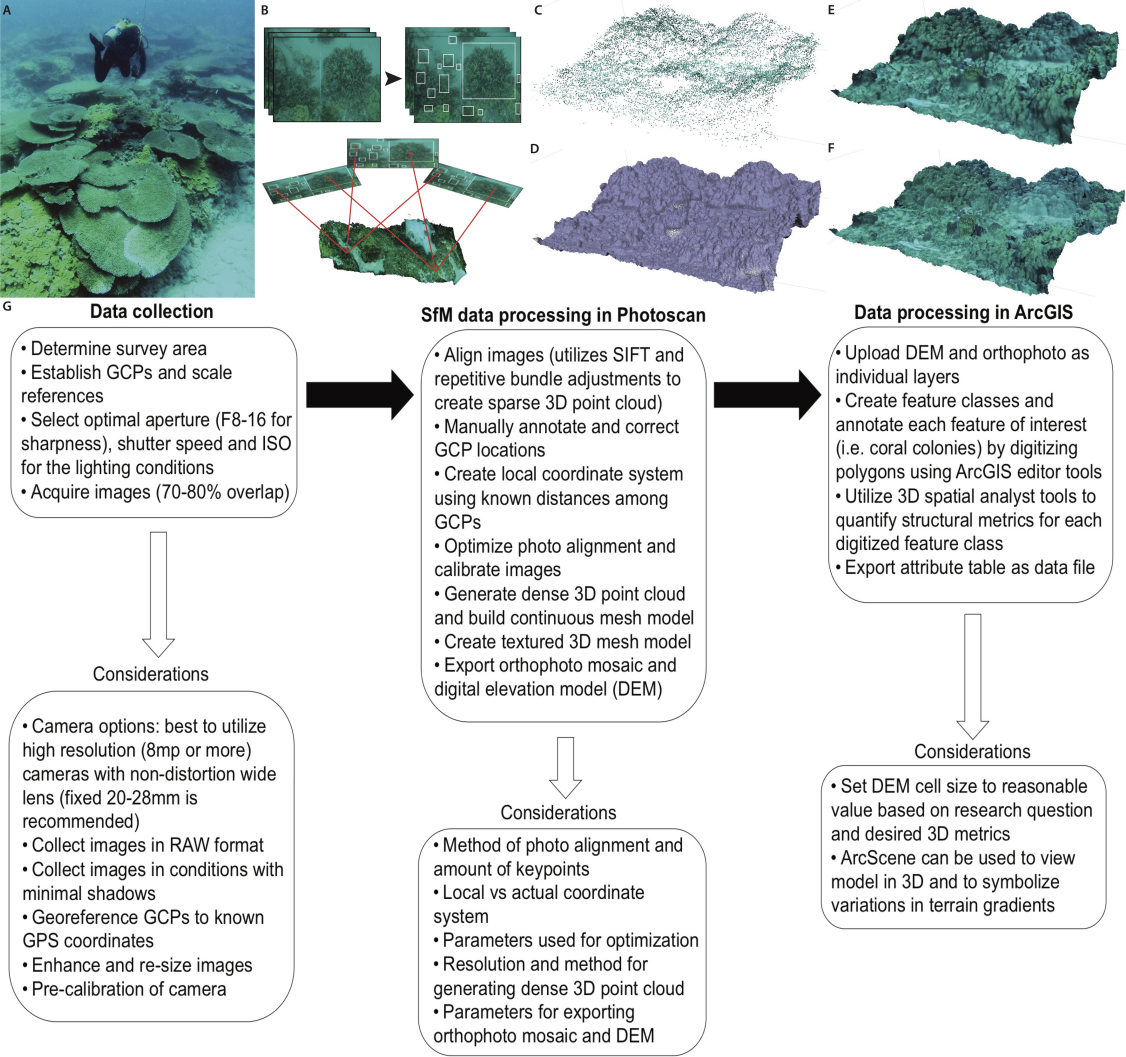
Primary steps of the SfM workflow for three-dimensional reconstruction of the benthic substrate. (A) Divers collect imagery while swimming above the coral reef substrate in a boustrophedonical pattern to enable 70–80% overlap among all images. (B) Scale invariant features (“keypoints”) are identified and extracted from the overlapping images. These keypoints are then matched and aligned to develop a photo mosaic of the substrate. (C) SfM software performs bundle adjustments to determine camera positions and construct a 3D point cloud which is then; (D) processed with soft-copy triangulation to reconstruct the scene geometry and create a solid 3D mesh that can be; (E) shaded or; (F) textured using the high-resolution photographs. (G) Flowchart summarizing the primary components and considerations for each step of the technique presented in this study.

3D models were constructed with the acquired imagery using Agisoft PhotoScan modeling software. PhotoScan was chosen because of the affordable cost, practical user interface, capability to determine camera parameters intrinsically and perform calibration, local processing, extremely detailed high-resolution 3D models, and the multiple exportable file formats compatible with geospatial software. There are a number of SfM programs that range from free software utilizing cloud processing to expensive professional-grade software that require powerful computing capabilities. Useful software reviews are available in order to determine the best program for the objectives and budget associated with a given research project (Remondino et al., 2012). The Agisoft modeling software is capable of generating the 3D digital surface model in three primary stages, (1) photo alignment (bundle adjustment), (2) geometry building, and (3) texture building. Photoscan aligns the series of photos from an individual survey using algorithms to automatically detect invariant features (“keypoints”), which overlap in the images, to create a system of geometrical projective matrices and determine the position and orientation of each camera position (Verhoeven, 2012; Westoby et al., 2012). The software constructs the 3D geometry on the 2D image plane using the extrinsic parameters that are calculated during the photo alignment (i.e., camera position and feature points) in conjunction with the intrinsic parameters and focal length of the camera that are extracted from the metadata of each image (Stal et al., 2012). The photo alignment (bundle adjustment) creates a sparse 3D point cloud that results from the projection and intersection of pixel rays from the different positions and oriented images in a 3D space. The *x*, *y*, *z* values from the GCPs are used to create a local coordinate system for accurate scaling and orientation of the point cloud, and at least 3 GCPs are needed for accurate 3D reconstruction (Verhoeven, 2012). Markers were annotated onto each of the GCPs using the Photoscan software. The location of each marker was manually reviewed and corrected in all 82 photos containing the GCPs (out of 441 total photos). This step required approximately 1-hour, and is dependent on both the amount of GCPs used and the number of photos containing the GCPs. Manually calibrating the location of GCPs using the Photoscan software ensures accurate interior and exterior orientation of the resulting model. After manually inputting the local coordinates of the GCPs, the model geometry is corrected by using the Photoscan optimize alignment tool. After correcting the interior and exterior orientation, the matching features are used to complete the final phase of the geometry building and generate a dense point cloud, which in turn can be used to build a continuous surface or mesh. The resulting mesh can be triangulated and rendered with the original imagery in order to build textured 3D mesh and create the final digital surface model (Fig. 1).

### Digital annotation of benthic features

**Figure 2:**
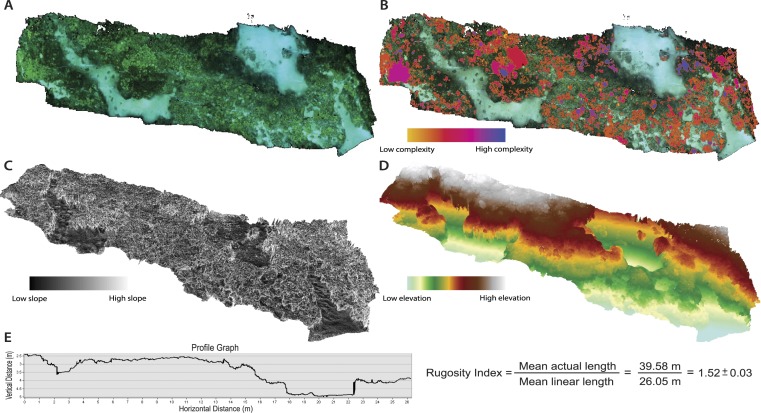
Orthophotos and digital elevation models (DEMs) produced with SfM photogrammetry techniques. (A) Orthophoto of the surveyed benthic habitat (28 × 6 m plot) at French Frigate Shoals provides a geometrically accurate image for annotation and topographic analyses. (B) Orthophoto with every coral colony individually annotated and symbolized to display the variability in surface complexity (3D/2D surface area). (C) DEM that represents percent slope of the surveyed habitat (maximum rate of change in value from one cell to its neighbors: tan *θ*∗100). (D) DEM representing elevational gradients throughout the surveyed habitat. (E) An example profile graph showing the DEM contour and the average Rugosity Index calculated from six profile graph permutations.

**Figure 3:**
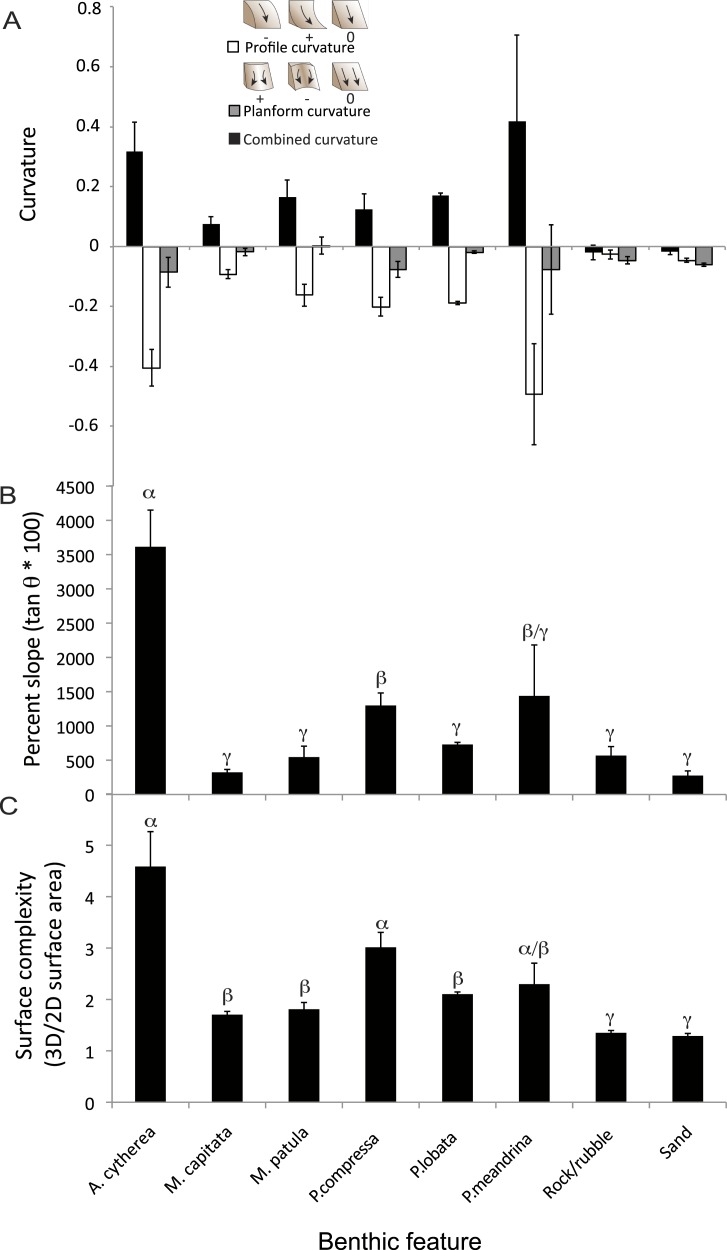
Structural metrics compared among the coral species and two substrate types present in the surveyed habitat. (A) Comparison of mean values (± S.E.) of combined (black), profile (white) and planform (grey) curvature among the benthic features. (B) Comparison of mean values (± S.E.) of percent slope among the benthic features. (C) Comparison of mean values (± S.E.) of surface complexity among the benthic features. *α*, *β*, and *γ* demark groupings identified as significantly different based on statistical analyses (ANOVA, *p* < 0.01).

The 2D orthophoto image file was exported from the Agisoft modeling software and uploaded into geospatial software for annotation and subsequent topographic analyses. The orthophoto provides a high-resolution photo-mosaic of the surveyed substrate, and is geometrically corrected such that each cell contains accurate 3D information and can be used for measurement of topographic parameters (Fig. 2A). The orthophoto was manually digitized in order to create unique polygon shapefiles for all individual living coral colonies (*Acropora cytherea, Montipora capitata, M. patula, Pocillopora meandrina, Porites compressa, and P. lobata*) and abiotic features (rock/rubble and sand) present in the surveyed benthic habitat. Benthic features were digitized using the ArcGIS (ArcMap 10.1; Environmental Systems Resource Institute, Redlands, California, USA) editor tools. A ‘feature class layer’ was created within ArcGIS, and each digitized polygon was assigned to a specific sub-type that represented each benthic feature in order to catalogue the benthic substrate. This post-processing procedure enabled spatial data to be quantified for each individual coral colony (2,290 total colonies) and abiotic feature (Fig. 3 and Table 1). Manually digitizing all features in the orthophoto required approximately 20-hours to complete. Annotating the orthophoto provided the means to compare topographic complexity data among all the living coral colonies and abiotic features that comprise the surveyed benthic habitat at French Frigate Shoals.

**Table 1:** Values of structural metrics compared among the coral species and two substrate types present at the French Frigate Shoals study site.

	Structural metrics					
Benthic component	Mean surface complexity (±S.E.)	Percent cover	Mean percent slope (±S.E.)	Combined curvature (±S.E.)	Profile curvature (±S.E.)	Planform curvature (±S.E.)
*A. cytherea*	4.59 ± 0.68	0.32%	3,614 ± 536	0.32 ± −0.09	−0.41 ± 0.06	−0.09 ± 0.05
*M. capitata*	1.71 ± .0.06	0.19%	323.3 ± 42	0.07 ± 0.02	−0.09 ± 0.01	−0.02 ± 0.01
*M. patula*	1.81 ± 0.13	0.11%	546 ± 159	0.16 ± 0.05	−0.16 ± 0.03	0.00 ± 0.02
*P. compressa*	3.02 ± 0.29	1.07%	1,299 ± 182	0.12 ± 0.05	−0.20 ± 0.03	−0.08 ± 0.02
*P. lobata*	2.12 ± 0.04	10.96%	733.7 ± 30.6	0.17 ± 0.01	−0.19 ± 0.01	−0.02 ± 0.00
*P. meandrina*	2.30 ± 0.41	0.03%	1,438 ± 744	0.42 ± 0.28	−0.49 ± 0.17	−0.08 ± 0.14
*Rock/rubble*	1.35 ± 0.05	74.75%	568 ± 131	−0.02 ± 0.02	−0.03 ± 0.01	−0.05 ± 0.01
*Sand*	1.29 ± 0.05	12.56%	275.5 ± 68	−0.02 ± 0.01	−0.05 ± 0.01	−0.06 ± 0.01

### Quantification of spatial properties

To quantify spatial properties pertaining to the configuration, conformation, contour, form, and shape of the coral reef environment, a digital elevation model (DEM) of the surveyed reef was exported from the Agisoft Photoscan modeling software into ArcGIS. The DEM exported from Photoscan can be uploaded to ArcGIS and matched to the same local coordinate system as the orthophoto. Within the ArcGIS software, the DEM is layered with the annotated orthophoto, and parameters relating to the 3D spatial characteristics of the reef can be quantified using spatial analysis tools in the ArcGIS program. 3D topographic features are quantified from the DEM by calculating the surface properties of cells in 3 × 3 windows. The values of the 3D metrics and overall spatial resolution are therefore sensitive to cell size, which can be set to any desired value using the ArcGIS software. It is important to note that the cell size should be set to an appropriate value based on the features being modeled. For this study, the raster cells of the DEM were set to 0.5-cm (1.5 × 1.5-cm window) in order to detect intricate structural differences among the various morphologies of the surveyed coral colonies. This component of the workflow must be adequately considered in order create a DEM with a spatial resolution that is appropriate for the desired ecological application. The following metrics were quantified as they have been shown to influence the key biological and ecological processes of coral reef ecosystems (Friedlander & Parrish, 1998; Kostylev et al., 2005; Pittman, Costa & Battista, 2009; Pittman & Brown, 2011). All metrics were quantified using tools within the ArcGIS topography software, with specific details provided below.

#### Linear rugosity and percent cover

Linear rugosity is calculated as the actual distance (accounting for changes in vertical surfaces along the reef contour) divided by the linear distance between two points (Luckhurst & Luckhurst, 1978; McCormick, 1994; Friedlander & Parrish, 1998). ArcGIS ‘path distance’ tool was used on the DEM to quantify a linear rugosity value for the surveyed reef plot by quantifying path distance across the across 6 different planes of the coral reef DEM, thus providing an average rugosity value for the surveyed area (Fig. 2). This tool quantified the path distance between start and end nodes on horizontal, vertical, and diagonal movement. Rugosity values were determined by comparing the path distance to the direct linear distance and an average was calculated for the surveyed reef plot (Fig. 2E). Percent cover was quantified by calculating the 2D surface area cover of each benthic feature in proportion to the overall 2D surface area of the surveyed reef plot.

#### Surface complexity

The 2D and 3D surface area was quantified using the ‘add surface information’ tool for all digitized polygons representing the benthic habitat. This tool is part of the ArcGIS spatial analysis toolbox and uses the orthophoto in conjunction with the DEM to calculate 2D and 3D surface area, as well as several other spatial properties. The ArcGIS editor function was used to calculate the ratio of 3D/2D surface area for each digitized polygon. This value of 3D/2D surface area was used as a 3D metric to represent ‘surface complexity’ for all benthic features.

#### Slope

Slope was calculated using the ‘slope’ tool in ArcGIS. This tool determines the maximum rate of elevational change from each cell to its neighbors in units of degrees. This tool fits a plane to the *z*-values of the 3 × 3 cell window around the processing or center cell. The slope value of this plane is calculated using the average maximum technique (Burrough & McDonell, 1998). The direction the plane faces is the aspect for the processing cell. A small slope values reflects flat terrain, while a large slope value infers a steep terrain. The output slope values were calculated as ‘percent slope,’ which is the rise divided by run (tangent *θ*) multiplied by 100.

#### Curvature

Analyzing the planform and profile curvature provided a metric for identifying areas of rapid changes in slope or aspect of the coral reef. The ‘curvature’ tool in ArcGIS was used to quantify the curvature of the DEM surface on a cell-by-cell basis, as fitted through that cell and its eight surrounding neighbors. Curvature is the second derivative of the surface, or the slope-of-the-slope. This metric represents the rate of change in surface curvature and quantitatively represents convexities and concavities, thus identifying distinct physical features in a topographic context (Pittman, Costa & Battista, 2009; Pittman & Brown, 2011). Two optional output curvature types are possible: the profile curvature is in the parallel direction of the maximum slope, and the planform curvature is perpendicular to the direction of the maximum slope. Large values of curvature represent complex terrain, and positive or negative values are indicative of either upwardly concave or convex surface in the vertical (profile) and horizontal (planform) direction relating to slope. Units of the curvature output raster, as well as the units for the optional output profile curve raster and output plan curve raster, are one hundredth (1/100) of the *z*-unit (0.5-cm). The values were adjusted to a 1-m scale in order to compare to other terrestrial and marine studies that primarily compute this metric from DEMs with a 1 m *z*-unit. ArcGIS multiplies the curvature values by −100 to give values in the approximate range [−1, 1] with a sign that ensures that positive curvature equates to convex forms and negative curvature equates to concave forms. The resulting values represent the radius of curvature, and can be understood as directly relating to the size of a circle or a sphere that just touches the surface at sampled points (De Smith, Goodchild & Longley, 2015). Curvature measures: cross-sectional, maximum and minimum curvature, are used to characterize surface features in order to identify peaks, ridges, passes, planar regions, channels and pits (De Smith, Goodchild & Longley, 2015). The ArcGIS software summarizes the descriptive statistics for each benthic feature, therefore providing one average value and corresponding level of variance (Table 1). This provides a useful method for assess curvature values, however statistical analyses cannot be performed outside of the ArcGIS software as values are not computed for each annotated polygon.

### Statistical analysis

Statistical analysis was performed to compare the 3D metrics of percent slope and surface complexity among all 2,290 coral colonies (*A. cytherea, M. capitata, M. patula, P. meandrina, P. compressa, and P. lobata)* and abiotic features (rock/rubble, sand) that were annotated within the surveyed reef plot. Data generated from the topographic analyses were transformed, if necessary, using log transformations, to meet the assumptions of normality and equal variance necessary for use of parametric statistical tests. Variation in mean values of percent slope and surface complexity were analyzed among the coral colonies and abiotic features using a multivariate analysis of variance (MANOVA). Since all features were analyzed in the same reef plot, a MANOVA was run initially in order to determine if collinearity existed among the variables that may increase the likelihood of Type I error. Further one-way univariate analysis of variance (ANOVA) and Tukey’s HSD post hoc tests were used as follow-up tests to the MANOVA in order to determine if statistical differences (*α* = 0.01) existed among the benthic features. All statistical tests were run using Minitab 15 (Minitab Inc., State College, Pennsylvania, USA) software.

## Results

The ground sample distance (resolution/pixel) of the resulting 3D model was 0.00109-m/pix with an error of 0.501 pix, thus our designated DEM cell size of 1.5-cm was well within the range of accuracy of the model. 100% of the images were matched with a median of 11715.7 keypoint matches per image and a total of 965,640 tie-points.

The SfM process renders textured 3D models in multiple file formats that serve as powerful visualization tools for assessing the dynamics of structural complexity in coral reef environments. Symbology gradients were applied to the DEMs in order to visualize variability in structural topography of the surveyed habitat for both percent slope and depth (Figs. 2C and 2D). These 3D models serve as a useful tool for visually representing physical characteristics, such as depth and slope, of the benthic habitat.

There were statistically significantly differences in the mean values of percent slope and surface complexity among the benthic features (biotic—corals, abiotic—rock/rubble and sand) of the reef at French Frigate Shoals (MANOVA, *F* = 22.27, *p* < 0.001). Further univariate analysis showed that percent slope, calculated as the tangent of *θ* multiplied by 100, was significantly greater in the coral, *A. cytherea*, than all other corals and abiotic features (ANOVA, *F* = 12.39, *p* < 0.001, Fig. 3B). Surface complexity, calculated as the ratio of 3D surface area to 2D surface area, was significantly greater in the corals exhibiting plating and branching morphologies (*A. cytherea*, *P. compressa*, and *P. meandrina*) compared to the encrusting and mounding corals (*M. capitata, M. patula, and P.* lobata), and significantly lower in the abiotic substrate (ANOVA, *F* = 30.63, *p* < 0.001, Fig. 3C). The values of combined, profile, and planform curvature varied among the biotic and abiotic benthic features. *A. cytherea* and *P. meandrina*, coral species with branching and plating morphologies, exhibited distinctly high values of both combined and profile curvature (Fig. 3A and Table 1). All coral colonies showed higher values of combined curvature compared to the abiotic features. Values of percent cover showed that the benthic habitat was predominantly comprised of abiotic features, and *P. lobata* was the dominant coral species (Table 1). The average Rugosity value of the surveyed habitat was 1.52 ± 0.03 S.E. (Fig. 2E).

## Discussion

This study utilized Structure for Motion (SfM) photogrammetry techniques in order to characterize the 3D topographic structure of a coral reef at French Frigate Shoals, an atoll located in the Papahānaumokuākea Marine National Monument. Development of cost- and time-effective methods for quantifying 3D characteristics of coral reefs is critically needed considering that for the past six decades ecologists have found multiple measures of complexity to be primary drivers of biodiversity in these ecosystems (Goreau, 1959; McCormick, 1994; Knudby & LeDrew, 2007; Walker, Jordan & Spieler, 2009; Dustan, Doherty & Pardede, 2013). Integration of the 3D products derived from SfM photogrammetry with geospatial software tools, as developed in this study, provides a novel method for accurately characterizing structural complexity of coral reef habitats for ecological applications. Because this process requires integration of a number of steps, multiple variables should be carefully considered when determining the feasibility and accuracy of this approach to suit the needs of each study. For example, camera and lens, calibration, software options, DEM scale, annotation, and environmental conditions are some of the many factors that can affect the resulting models and quantification of 3D metrics (Bianco et al., 2011; McCarthy & Benjamin, 2014). Our approach was optimized for working in a remote field location with the goal of conducting a rapid survey with minimal equipment that would facilitate 3D reconstruction to enhance on-going studies of coral health and community structure.

We quantified 3D metrics that are informative of topographic structural complexity of a coral reef. The 3D structure of an environment directly affects important ecological parameters such as habitat provisioning and light availability and can therefore strongly influence ecosystem function—the biological, geochemical, and physical processes and components occurring within an ecosystem that drive biomass production, energy flux and other ecological services (Crowder & Cooper, 1982; Alvarez-Filip et al., 2009; Graham & Nash, 2013). Despite the known importance of structural complexity for ecosystem function, the difficulty in collecting 3D data has meant that the majority of ecological studies have been limited to utilizing two-dimensional (2D) planar survey techniques. While reducing dimensionality in sampling is beneficial for the cost and time constraints associated with ecological surveys, it limits the quality of data and is incapable of detecting specific 3D structural characteristics that are drivers of habitat facilitation and key ecological services (Goatley & Bellwood, 2011). The 3D metrics such as slope, curvature, and surface complexity of corals not only affects the diversity and abundance of species hosted by the coral community, but also may influence hydrodynamics and the amount of light irradiance received by photosynthetic organisms (Kostylev et al., 2005; Guinan et al., 2009; Pittman, Costa & Battista, 2009; Pittman & Brown, 2011). Previous studies have found that the specific 3D metrics used in our study were important predictors of vegetation distribution on terrestrial landscapes and influenced the distribution, biomass and diversity of vertebrate and invertebrate species in marine systems (Friedlander & Parrish, 1998; Kostylev et al., 2005; Pittman & Brown, 2011). Our study obtained these 3D metrics from 2,290 individual living coral colonies and surrounding abiotic features. We found that plating and branching corals (*Acropora cytherea, Porites compressa*, and *Pocillopra meandrina*) exhibited the greatest levels of surface complexity, slope, and curvature (Fig. 3 and Table 1). Furthermore, all three of these more complex species yielded large values for profile and planform curvature, indicating the coral colony surfaces are primarily concave in the vertical plane and convex in the horizontal plane (Fig. 3A). Such combination of concave and convex structure likely plays a substantial role in creating more microhabitat space per unit area for organisms using corals as shelter, and therefore may support more diversity, species richness and abundance of organisms (Alvarez-Filip et al., 2009; Graham & Nash, 2013). Among the coral species found to be structurally complex, *A. cytherea* had the greatest values of slope (Fig. 3B), which is indicative of substantial transitions in terrain surface. The large slope values are derived from the distinct plating morphology of this species, and are associated with the overhanging of the plates. The data presented here suggests that other reefs located at French Frigate Shoals with coral communities dominated by *A. cytherea, P. meandrina, and P. compressa* provide the most complex habitat and potentially support higher levels of diversity and productivity. In summary, utilizing the 3D approach described here can help to identify key coral species that contribute to high levels of productivity and diversity in coral reef ecosystems. Furthermore, the 3D characterization can track the effects of environmental perturbations on this ecosystem. Corals exhibiting complex branching, plating and corymbose morphologies, such as *Acropora, Pocillopora*, and branching *Porites* are generally more susceptible to disturbance than slow-growing corals that exhibit massive morphologies (Gates & Ainsworth, 2011; Woesik et al., 2011). Quantifying the relationship between the 3D structural characteristics of corals and assemblages of epibenthic organisms can help to elucidate potential changes to ecological functionality associated with local and global stressors that cause mortality and loss of coral cover.

In addition to the values representing 3D structural complexity, traditional benthic parameters including relative cover of coral species and abiotic substrate types and rugosity were analyzed using the orthophoto and DEM with the ArcGIS topographic software. Analyzing relative cover provides a useful construct perspective from which to assess reef structure and complexity. While the corals, *A. cytherea, P. compressa, and P. meandrina* were the most structurally complex, they only occupied a total of 1.42% of the surveyed space. On the other hand, *Porites lobata* was the dominant coral, occupying 10.96% of the benthos, thus overall biotic reef structure is predominantly driven by *P. lobata* at this site. Abiotic substrate (rock, rubble, sand) occupied 87.31% of the benthos, therefore indicating the habitat at this particular site is primarily composed of abiotic features. Assessing the 3D structural dynamics of a benthic habitat in conjunction with percent cover is critical for characterizing the site in an ecological context. Determining how each feature is distinctly different in terms of habitat structure is equally as important as identifying dominant features, as the dominant features will likely have the largest impact on associated species assemblages. It is critical to determine the morpho-functional groups of corals in order to understand how shifts in coral dominance will induce changes in biodiversity and ecosystem services (Alvarez-Filip et al., 2011). Rugosity, measured as the ratio of a straight line transect to a flexible chain draped over the substrate, has been the universally accepted measure of habitat complexity for several decades (Risk, 1972; Friedlander & Parrish, 1998; Dustan, Doherty & Pardede, 2013). While this technique is simple and inexpensive, the chain can easily tangle as well as disrupt the benthic habitat, and this method only captures a crude measure of complexity at a small scale. The SfM technique described in this paper allows for a rugosity profile to be virtually measured along any segment of the surveyed area (Fig. 2E). Quantifying multiple permutations of rugosity provides a much more accurate value of this metric, and is far more efficient than a single-chain extrapolation.

The utility of the SfM photogrammetry and associated geospatial analysis of reef complexity described in this paper has wide applications in coral reef research and management. Future studies can utilize these metrics to decipher the specific elements of habitat complexity that drive biodiversity, thus enhancing the ability to model future changes in ecosystem functionality associated with changes or loss in coral structure. Long-term monitoring of coral reef habitats can also be enhanced with SfM photogrammetry techniques. This method not only provides visual tools for assessing changes over time, such as the orthophoto and photo-mosaic, but the point clouds can also be compared across temporal scales in order to precisely quantify changes occurring in the volumetric and structural properties of corals. This will be critical for accurately investigating parameters such as coral biomass and habitat availability for reef fishes and invertebrates, all of which plays a vital role in coral reef productivity (Luckhurst & Luckhurst, 1978; Abdo et al., 2006; Wilson, Graham & Polunin, 2007). Examining changes in coral biomass and structure is also essential for accurately determining how coral reefs will respond to exponentially increasing global stressors, such as climate change and ocean acidification (Goatley & Bellwood, 2011; Graham & Nash, 2013). It is our hope that this study will provide a methodological platform for future studies to accurately quantify the 3D properties of coral reefs to improve our collective understanding of these important ecosystems.

## Supplemental Information

10.7717/peerj.1077/supp-1 Supplemental Information 1 Raw data Raw data pertaining to the structural metrics analyzed in this study.

## Additional Information and Declarations

### Field Study Permissions

The following information was supplied relating to field study approvals (i.e., approving body and any reference numbers): The National Oceanic and Atmospheric Administration (NOAA), the US Fish and Wildlife Service (FWS), and the State of Hawaiʻi (collectively, the Co-Trustess) approved issuance of permit number PMNM-2012-031 to conduct activities within the Papahānaumokuākea Marine National Monument.
